# The Safety of a High-Flow Nasal Cannula in Neuromuscular Disease Patients with Acute Respiratory Failure: A Retrospective Case-Series Study

**DOI:** 10.3390/jcm12186061

**Published:** 2023-09-19

**Authors:** Federico Lionello, Francesco Lapia, Beatrice Molena, Andrea Padoan, Sara Lococo, Giovanna Arcaro, Gabriella Guarnieri, Andrea Vianello

**Affiliations:** 1 Department of Cardiac, Thoracic, Vascular Sciences and Public Health, University of Padova, Via Giustiniani 2, 35128 Padova, Italy; federico.lionello@aopd.veneto.it (F.L.); beatrice.molena@unipd.it (B.M.); sara.lococo@aopd.veneto.it (S.L.); giovanna.arcaro@aopd.veneto.it (G.A.); gabriella.guarnieri@unipd.it (G.G.); 2 Department of Internal Medicine and Medical Therapy, University of Pavia, Piazza Golgi 19, 271000 Pavia, Italy; francesco.lapia01@universitadipavia.it; 3 Department of Medicine, University of Padova, Via Giustiniani 2, 35128 Padova, Italy; andrea.padoan@unipd.it

**Keywords:** non-invasive ventilation, high-flow nasal cannula, acute respiratory failure, neuromuscular disease

## Abstract

(1) Background: Although Non-Invasive Ventilation (NIV) is effective in preventing mortality and endotracheal intubation in patients with Acute Respiratory Failure (ARF) linked to a neuromuscular disorder, its efficacy can be affected by patient intolerance. A High-Flow Nasal Cannula (HFNC) appears to have a significant advantage over NIV as far as patient tolerance is concerned. The aim of the study was to investigate HFNC’s safety profile in a group of consecutive Neuromuscular Disease (NMD) patients intolerant to NIV who were admitted to an Intermediate Respiratory Care Unit (IRCU) for ARF. (2) Methods: The clinical course of nine NMD patients intolerant to NIV and switched to HFNC was reported. HFNC was provided during daytime hours and NIV during the night-time to the NIV-intolerant patients. HFNC was utilized 24 h a day in those patients who were intolerant of even nocturnal NIV. (3) Results: HFNC was simple to use and it was well tolerated by all of the patients. Three out of nine patients experienced treatment failure, consisting of the need for ETI and/or death during their IRCU stay. The remaining 6 had a favorable outcome. Treatment failure was linked to the utilization of HFNC 24 h a day. (4) Conclusion: HFNC during the daytime hours, together with nocturnal NIV, seems to be a safe therapeutic approach for NMD patients with ARF. A round-the-clock use of HFNC tends to be linked to a high likelihood of failure.

## 1. Introduction

Acute Respiratory Failure (ARF), which is a common occurrence in teenage and adult patients suffering from Neuromuscular Disease (NMD), is a primary cause of mortality in this population [1]. Most physicians agree that Non-Invasive Ventilation (NIV) can be an effective first-line therapy to reduce the risk of mortality in NMD patients with ARF, especially in those presenting hypercapnic respiratory acidosis, as it can reduce the need for Invasive Mechanical Ventilation (IMV) and the risk of related complications, in particular Ventilator Associated Pneumonia (VAP) [2,3,4]. 

NIV efficacy can nevertheless be partially affected by patient intolerance, especially on the part of infants and young children who may be agitated or uncooperative [5]. Signs of NIV intolerance can appear at the onset of treatment or at a later date, as NIV-related complications worsen. The development of mask-related skin abrasions or necrosis appears to be one of the primary factors linked to NIV intolerance [6]. Poor tolerance in NMD patients has also been associated with gastric and/or colonic distension, or the accumulation of bronchial secretions due to the patient’s inability to cough forcefully [2,7,8]. A Non-Invasive Respiratory Support, which may be used in those cases as an alternative to NIV to reduce the need for Endotracheal Intubation (ETI) and IMV, becomes important.

A High-Flow Nasal Cannula (HFNC) delivers heated, humidified air and oxygen via wide-bore nasal cannulas at a prescribed fraction of inspired oxygen (FiO_2_) and high-flow rates. Some studies have recently reported improved patient comfort during HFNC therapy, which seems to have a significant advantage over NIV as far as patient tolerance is concerned [9]. Those studies have concluded that HFNC could be used as an alternative to NIV within the context of an integrated “non-invasive ventilatory strategy” to manage subjects with ARF [10]. Yet despite its promising potential, HFNC has not gained any real popularity among physicians managing exacerbated NMD patients with ARF, probably because of the risk of harmful consequences. Indeed, some experts have warned against providing supplemental oxygen to NMD individuals in an acute setting as it could reduce the drive to breathe leading to potentially fatal CO_2_ retention [11,12]. To date, sporadic case-reports only described the use of HFNC in patients with ARF of neuromuscular origin. In particular, HFNC was well tolerated in an exacerbated Amyotrophic Lateral Sclerosis (ALS) patient with hypercapnic ARF (hARF), whose response to treatment was similar to the one generally expected for NIV [13]. Moreover, HFNC appeared to be better tolerated than NIV in a patient with ARF consequent to immune-related myasthenia gravis [14]. By contrast, nighttime provision of HFNC at 20 and 50 L/min (without additional O_2_ supplementation) was found to be poorly tolerated in a group of 17 clinically stable patients with genetically proven NMD [15].

The current study was carried out with the intention of retrospectively evaluating HFNC’s safety profile in a series of consecutive NMD patients with ARF showing poor NIV tolerance who were admitted to an Intermediate Respiratory Care Unit (IRCU). 

## 2. Methods

This single center, retrospective case-series study was conducted at the IRCU of the University of Padua Medical Center between 1 January 2020 and 31 March 2023. All of the participants signed general consent statements authorizing the use of their de-identified clinical data for research, analysis, and reporting purposes; the data were anonymized by assigning a de-identified code to each file. The need for ethical approval was waived by the local Ethics Committee in view of the fact that the study was retrospective and not prepared according to a research project. The study was carried out in accordance with the Declaration of Helsinki of 1975.

### 2.1. Patients

The short-term outcomes of nine consecutive NMD patients who were switched to HFNC as a result of partial or full NIV intolerance after being admitted to the IRCU during the study period were investigated. The patients’ NIV intolerance was considered to be partial or full if NIV needed to be discontinued during the daytime only or also during the night, respectively. The clinical and physiologic parameters of these patients were consistent with ARF at the time they were admitted to the IRCU [16].

The following demographic and clinical information was collected from the patients: age, gender, BMI, smoking habit, presence of a percutaneous gastrostomy (PEG) tube, type of NMD, comorbidities. The following parameters at the time of the patients’ admission to the IRCU were collected: the diagnosis related to ARF, Heart Rate (HR), Respiratory Rate (RR), white blood cell count, serum C-reactive protein (CRP), arterial PaO_2_, PaCO_2_, and pH during spontaneous breathing on room air or supplemental oxygen, and arterial oxygen tension (PaO_2_) to inspired oxygen fraction (FiO_2_) ratio (PaO_2_/FiO_2_) (Table 1). Arterial blood gas (ABG) levels registered at the time the patient was switched to HFNC were also collected. ABG data 2 h and 12 h after the switch to HFNC, as well as the times between initiating NIV and the switch to HFNC, were collected (Table 2). 

### 2.2. Interventions

HFNC therapy was delivered using an AIRVO2 respiratory humidifier (Fisher & Paykel Healthcare, Auckland, New Zealand) with an integrated flow generator able to adjust FIO_2_ (between 0.21 and 1.0) and to deliver an air/oxygen mixture at flow rates of up to 60 L/min. The gas mixture (at 37 °C) is routed through a circuit via large-bore bi-nasal prongs. HFNC was initially used at a 60 L/min gas flow rate and a FIO_2_ of 1.0; it was rapidly adjusted to release the minimum FIO_2_ necessary to maintain a SaO_2_ ≥ 92% at the maximum gas flow rate tolerated by the patient. The patients demonstrating discomfort, agitation or unwillingness to accept NIV were initially offered HFNC during the daytime and NIV during the night. After a trial, those patients who were intolerant of even NIV during the night were offered HFNC for the entire 24 h period. HFNC treatment was administered as long as conventional oxygen therapy was able to achieve a SaO_2_ of ≥92%. The NMD patients were considered intolerant to HFNC in those cases where it became necessary to terminate therapy due to discomfort (i.e., paradoxical suffocation and/or “chest pressure”), agitation or uncooperativeness. Adverse events (AEs) related to HFNC-utilization, including treatment failure leading to ETI or death, barotrauma, epistaxis, and/or nose irritation were recorded. 

NIV was delivered using a portable ventilator set on the pressure support (PS) ventilation mode and a full-face mask. PS was initially titrated to a moderate tidal volume (6–8 mL/kg of ideal body weight); the ventilator setting was then readjusted depending on the ABG data in an effort to ensure a satisfactory, although not necessarily optimal, gas exchange, while nevertheless still protecting the lungs from the risk of Ventilator-Induced Lung Injury (VILI). Supplemental oxygen was added to the ventilator circuit; the oxygen flow rate was set to achieve an arterial SaO_2_ ≥ 92%. AEs related to NIV utilization, including treatment failure leading to ETI or death, barotrauma, aspiration pneumonia, gastric and/or colonic distension, and skin lesions were recorded.

With the exception of those patients who had previously declared that they did not wish to be intubated, emergency ETI was performed in the event any of the following occurred during the application of HFNC: respiratory arrest; loss of consciousness with respiratory pauses, gasping for air; HR < 50 bpm with loss of alertness; hemodynamic instability with systolic blood pressure < 70 mmHg. [17] Non-emergent ETI was performed in the event of HFNC’s inability to maintain a SaO_2_ ≥ 92%, clinically important CO_2_ retention, and/or the onset of a severe risk of inhalation. Electrocardiography, pulse oximetry, invasive and/or non-invasive blood pressure, and RR were continuously monitored in all of the patients.

### 2.3. Outcome Measures and Statistical Analysis 

In accordance with the guidelines of the European Medicines Agency (EMA) for the evaluation of drug clinical safety, [18] HFNC safety was assessed on the basis of the number of AEs that occurred. In particular, the number of treatment failures, consisting of the need for ETI and/or death during an IRCU stay, was considered. Minor AEs were also recorded, including barotrauma, epistaxis, and/or nose irritation. The course of PaCO_2_ during the 12 h period after the patient was switched to HFNC, and the number of patients who developed clinically important CO_2_ retention at the 2 h and 12 h follow-up evaluations, were assessed to evaluate the risk of CO_2_ retention. The time interval chosen for assessing CO_2_ retention was in line with previous studies investigating oxygen-induced hypercapnia in an acute setting [19,20]. Clinically important CO_2_ retention was defined as a rise in PaCO_2_ > 7.5 mmHg [20]. The outcomes were censored on 31 May 2023 with regard to those patients still hospitalized on that day.

The results are expressed as median and interquartile ranges (IRQ). The Kolmogorov–Smirnov test was used to check the normality of the data distribution. All the calculations were carried out using Stata (Statacorp, Lakeway drive, TX, USA). 

## 3. Results

**Figure 1:**
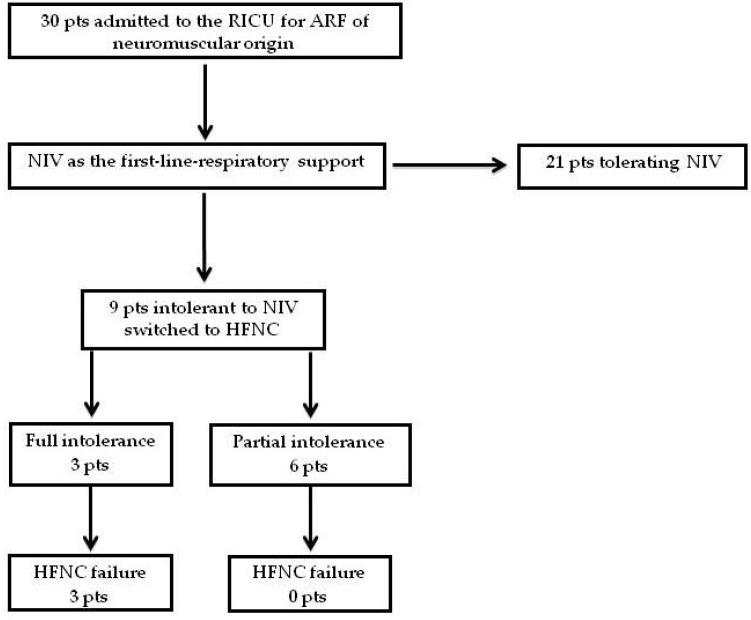
Study profile (HFNC = High Flow Nasal Cannula; NIV = Non-Invasive Ventilation; Pts = Patients).

Thirty patients with a primary diagnosis of NMD were admitted to our IRCU for ARF during the study period; they were all administered NIV as the first-line ventilatory intervention. Nine of the patients (30%) were intolerant to NIV (the intolerance was partial in six cases and full in three); they were switched to HFNC. Causes of NIV intolerance are outlined in Table 2. All nine were considered eligible to participate in our retrospective study. Twenty-one of the patients tolerated NIV (Figure 1). Baseline demographic and clinical characteristics, and clinical and laboratory data on IRCU admission of the patients who tolerated NIV well, are reported as Supplementary Materials (Table S1).

The anthropometric, clinical, pulmonary function and ABG data on IRCU admission and the clinical outcomes of each patient receiving HFNC are outlined in Table 1. Severe respiratory infection (pneumonia or bronchiectasis exacerbation) was the cause of acute decompensation in 7 out of the 9 intolerant-to-NIV patients.

At the time they were admitted to the IRCU, four of the intolerant patients were normocapnic, four showed moderate CO_2_ retention, and one showed severe CO_2_ retention [PaCO_2_: 54.1 (33.9−74.0) mmHg] [21]. The switch to HFNC was carried out 25 (1−36) hours after NIV was initiated. Most of the patients showed moderate hypercapnia at the time they were switched [PaCO_2_: 56.6 (38.7−68.1) mmHg] (Table 2).

HFNC was easy to set up and well tolerated by all of the patients. Six patients were prescribed HFNC during the daytime and NIV during the night-time. The other three patients were prescribed HFNC throughout the 24 h period. 

Following a switch to HFNC, three patients experienced treatment failure. Importantly, all of them had been switched to 24-hour-a-day HFNC therapy: two developed swallowing difficulties and were intubated due to the high risk of inhalation, and a patient rapidly developed severe pneumonia and died as a result of severe respiratory acidosis, having expressly requested not to be intubated. The remaining 6 patients had a favorable outcome. No other minor AEs were recorded. 

PaCO_2_ values 2 h after treatment was initiated were not significantly changed compared to baseline values [55.6 (45.6−64.8) vs. 56.6 (38.7−68.1) mmHg; *p* = 0.9544]. At that time, HFNC was used at a 60 (30−60) L/min gas flow rate and a FIO_2_ of 0.4 (0.21−0.73). None of the patients showed signs of clinically important CO_2_ retention at the 2 h and 12 h follow-up evaluations. 

## 4. Discussion

This report presents retrospective data collected to examine HFNC’s safety profile in nine NMD patients who were switched from NIV when they showed signs of intolerance or developed treatment-related complications. An analysis of the study’s results showed that three out of the nine patients experienced treatment failure. Since six out of the nine intolerant patients showed CO_2_ retention at the time they were switched, we hypothesize that a reduced ability of HFNC in reversing the physiological and mechanical derangements associated with acute hypercapnia may have been a major determinant of the adverse outcome. Unlike NIV, HFNC did not prove, in fact, to be effective in significantly increasing tidal volume and alveolar ventilation in individuals with ARF [22,23] although it may partially reverse hypercapnia by clearing the upper airways of expired air, which reduces anatomic dead space and makes ventilation more efficient [24]. Consistent with this hypothesis, PaCO_2_ values did not improve over the first 12 h following HFNC application. Treatment failure in the patients studied seemed, nevertheless, to be specifically linked to the round-the-clock use of HFNC: indeed, the three patients who required ETI or died during their stay in the IRCU had declined nocturnal NIV and chose to undergo continuous 24-hour-a-day HFNC treatment. By contrast, the subjects who received HFNC during the daytime and NIV during the night were discharged from the IRCU without any complications. We can assume that nocturnal assisted ventilation prevented the patients’ clinical deterioration by reversing sleep-related hypercapnia: indeed, the reduction of profound hypercapnia during sleep via nocturnal intermittent positive pressure ventilation is associated with an improvement in daytime ventilation and ABG values in NMD patients [25].

Importantly, daily use of an HFNC was well accepted by our patients as it does not need to be removed during oral hygiene care or while patients are talking, eating, or drinking.

The high tolerance rate towards HFNC confirms previous data showing that it is more effective than NIV in decreasing respiratory discomfort in subjects with ARF [26]. This enhanced tolerance can also be explained by the fact that HFNC reduces the risk of claustrophobic feelings or of skin lesions and/or mucous encumbrances associated with NIV use [27].

The study’s limitations include the small number of patients investigated and its retrospective nature, which may have caused a significant bias. However, all clinical studies examining patients with rare diseases such as NMD tend to present these limitations [28]. Moreover, as the study was conducted in a single center, the generalizability of its results is, of course, questionable.

Despite these important limitations, an analysis of the study’s data shows that:

- Daytime HFNC combined with nocturnal NIV seems to be a safe treatment strategy in NMD patients with ARF showing partial intolerance to NIV linked to treatment-related complications;
- A round-the-clock use of HFNC in ARF secondary to NMD tends to be linked to a high likelihood of failure;
- Whenever HFNC is provided to NMD patients, PaCO_2_ levels need to be closely monitored.

Given its simplicity, the cost efficiency of the equipment involved, and a greater patient tolerance than with NIV, adequately resourced clinical trials are needed to evaluate the efficacy of and conditions for using HFNC in exacerbated NMD patients.

## Figures and Tables

**Table 1:** Baseline demographic and clinical characteristics, clinical and laboratory data on Intermediate Respiratory Care Unit admission and clinical outcomes of patients switched to High Flow Nasal Cannula.

Patient, No.	1	2	3	4	5	6	7	8	9
**Baseline demographic and clinical data**									
Age, years	61	30	73	53	21	56	34	45	34
Gender	F	M	M	F	M	M	M	M	F
Body mass index, kg/m^2^	20.2	14.6	-	30.5	30.1	-	15	-	31.2
Smoking habit	Ex-smoker	Non-smoker	Non-smoker	Non-smoker	Non-smoker	Ex-smoker	Non-smoker	Non-smoker	Non-smoker
PEG tube	yes	no	no	no	no	no	no	yes	no
Type of NMD	ALS	CM	ALS	DM1	DMD	Type II SMA	Unspecified myopathy	ALS	AP
Comorbidity	Breast cancer	Testicular seminoma	-	Calculous cholecystitis	OSA	CHF diabetes	Hypertension	-	AATD
**Clinical, laboratory and ABG data on IRCU admission**									
Diagnosis related to ARF	Pneumonia	Pneumonia	Pneumonia	Interstitial pneumonia	PNX	Undernutrition	Pneumonia	Pneumonia, Atelectasis	Bronchiectasis exacerbation
HR, beats/min	91	79	120	69	100	83	102	105	62
RR, breaths/min	25	17	23	-	20	-	15	-	20
White blood cell count, ×10^9^/L	4.09	14.84	12.02	7.09	7.43	7.21	9.08	17.9	7.7
Serum CRP, μg/ml	8.67	205	102	5.48	20.04	13	3.18	94	0,6
PaO_2_, mmHg	80.9 *	57.4	167 *	64.3	57.5	56.2	81	132 *	92.8 *
PaCO_2_, mmHg	41.8	41.3	74	54.1	57.4	60.7	60	44.5	33.9
pH	7.43	7.47	7.32	7.39	7.38	7.28	7.45	7.45	7.44
PaO_2_/FiO_2_, mmHg	155.6	239.1	208.7	207.4	273.8	175.6	289.2	368.1	386.7
**Clinical outcomes**									
	ETI, trach, discharged	Discharged	ETI, trach, discharged	Discharged	Discharged	Discharged	Discharged	Died	Discharged

* Supplemental O_2_. AATD = Alpha 1 Antitrypsin Deficiency; ABG = Arterial Blood Gas; AHF = Acute Heart Failure; ALS = Amyotrophic Lateral Sclerosis; AP = Axonal Polyneuropathy; CHF = Chronic Heart Failure; CM = Congenital Myopathy; DM1 = Myotonic Dystrophy Type 1 DMD = Duchenne’s Muscular Dystrophy; HFNC = High Flow Nasal Cannula; IRCU = Intermediate Respiratory Care Unit; NIV = Non-Invasive Ventilation; NMD = Neuromuscular Disease; OSA = Obstructive Sleep Apnea; PEG = Percutaneous Gastrostomy; PNX = Pneumothorax; Trach = tracheostomy.

**Table 2:** Causes of Non-Invasive Ventilation intolerance, High Flow Nasal Cannula use and PaCO_2_ outcomes.

Patient, No.	1	2	3	4	5	6	7	8	9
Cause of NIV intolerance	Claustrophobia	Uncooperative	Agitation	Difficulty eating	Agitation	Difficulty eating	Difficulty eating	Dry mouth	Thoracic pain
Time to switch, h	0.5	0.5	1	15	48	36	25	64	25
HFNC use	24 h a day	Daytime	24 h a day	Daytime	Daytime	Daytime	Daytime	24 h a day	Daytime
PaCO_2_ at switch time, mmHg	43	38.7	68.1	59.2	65.8	56.5	62.5	56.6	41
PaCO_2_ at 2 h interval, mmHg (HFNC flow rate, L/min—FIO_2_)	41.3 (60–0.6)	45.6 (50–0.4)	67 (40–0.4)	51 (60–0.4)	65.9 (30–0.3)	60.4 (60–0.73)	64.8 (60–0.21)	55.6 (60–0.3)	36.9 (60–0.38)
PaCO_2_ at 12 h interval, mmHg (HFNC flow rate, L/min—FIO_2_)	46.6 (60–0.52)	39.1 (50–0.3)	61 (NA)	51 (60–0.8)	64 (30–0.3)	57.1 (NA)	59.3 (60–0.21)	41.5 (NA)	33.6 (50–0.27)

HFNC = High Flow Nasal Cannula; NA = Not available; NIV = Non-Invasive Ventilation.

## Data Availability

The clinical and respiratory function data that support the findings of this study are available at https://intranet.sanita.padova.it at request of the interested party.

## Supplementary Materials

The following supporting information can be downloaded at: https://www.mdpi.com/article/10.3390/jcm12186061/s1, Table S1: Baseline demographic and clinical characteristics, and clinical and laboratory data at Intermediate Respiratory Care Unit admission of patients who tolerated Non-Invasive Ventilation well.
