# Supplementary material for: The Safety of a High-Flow Nasal Cannula in Neuromuscular Disease Patients with Acute Respiratory Failure: A Retrospective Case-Series Study
Source: J Clin Med. 2023 Sep 19;12(18):6061. doi: 10.3390/jcm12186061 (PMC10531616; doi:10.3390/jcm12186061)
Supplement: Supplementary file 1 [file jcm-12-06061-s001.zip › jcm-2599956-supplementary.pdf]

Baseline demographic and clinical characteristics, and clinical and laboratory data at Intermediate Respiratory Care Unit admission of patients who tolerated Non-Invasive Ventilation treatment. Six patients were not included for incomplete clinical data.

Supplementary Table S1. Baseline demographic and clinical characteristics, and clinical and laboratory data at Intermediate Respiratory Care Unit admission of patients who tolerated Non-Invasive Ventilation well.

**Baseline demographic and clinical data**		
Age, years (median and IQR)	58 (39-70)	
Female, n (%)	2 (13.33)	
Body mass index, kg/m^2 (median and IQR)	21.6 (18.6-26.1)	
Pts previously administered PEG, n. (%)	2 (13.33)	
Type of NMD, n (%)		
o ALS	8 (53.3)	
o DMD	3 (20.0)	
o DM1	1 (6.67)	
o CM	1 (6.67)	
o CMD	1 (6.67)	
o BMD	1 (6.67)	
o CMT	0 (0.00)	
o SMA	0 (0.00)	
o AP	0 (0.00)	
Pts with comorbidities, n (%)		
o cardiac disease (cardiac arrhythmia, previous MI, angina pectoris, and/or CHF)	4 (26.6)	
o metabolic disorder (diabetes, obesity)	3 (20.0)	
o psychiatric disorders	3 (20.0)	
o hemato-oncology disease	0 (0.00)	
o immunologic disorders	1 (6.67)	
o respiratory disease (asthma, COPD)	0 (0.00)	
o chronic renal failure	0 (0.00)	
**Clinical, laboratory and ABG data on IRCU admission**		
Diagnosis related to ARF, n (%)		
o Pneumonia	6 (40.00)	
o Gastric/colonic distention	2 (13.33)	
o OSA	2 (13.33)	
o Fatigue	2 (13.33)	
o Bronchiectasis exacerbation	0 (0.00)	
o Pneumothorax	0 (0.00)	
o Malnutrition	0 (0.00)	
o Acute heart failure	1 (6.67)	
o Acute bronchitis	1 (6.67)	
o Lung Cancer	1 (6.67)	
Heart rate, beats/min (median and IQR)	82 (76-103)	0.8114
Respiratory rate, breaths/min (median and IQR)	15 (14.5-17)	0.0777
White blood cell count, x 10^9/L (median and IQR)	7.16 (5.99-10.96)	0.2967
Serum C-reactive protein, mg/dL (median and IQR)	7.55 (2.5-55)	0.3247
PaO_2, mmHg (median and IQR)	68 (63.9-80)	0.5258
$PaCO_2$, mmHg (median and IQR)	49.7 (39.5-55.5)	0.4929
Arterial pH (median and IQR)	7.42 (7.36-7.45)	0.8811
SaO_2, % (median and IQR)	96 (94-99)	0.7717
PaO_2/FiO_2, mmHg (median and IQR)	312.4 (268.1-323.8)	0.1330

ABG= Arterial Blood Gas; ALS= Amyotrophic Lateral Sclerosis; AP= Axonal Polyneuropathy; BMD= Becker Muscular Dystrophy; CHF= Chronic Heart Failure; CM= Congenital Myopathy; CMD= Congenital Muscular Dystrophy; CMT= Charcot-Marie-Tooth disease; COPD= Chronic Obstructive Pulmonary Disease; CRP= C-Reactive Protein; DM1= Myotonic Dystrophy Type 1; DMD= Duchenne's Muscular Dystrophy; ETI= Endotracheal Intubation; HFNC= High Flow Nasal Cannula; IRCU= Intermediate Respiratory Care Unit; MI= Myocardial Infarction; NIV= Non-Invasive Ventilation; OSA= Obstructive Sleep Apnea; PaO_2/FiO_2= Arterial Oxygen Tension to Inspired Oxygen Fraction Ratio; PEG= Percutaneous Gastrostomy; SaO_2= arterial oxygen saturation.
